# A pilot study about the development and characterization of a Roux en Y gastric bypass model in obese Yucatan minipigs

**DOI:** 10.1038/s41598-021-98575-8

**Published:** 2021-10-12

**Authors:** Damien Bergeat, S. Blat, Y. Gautier, S. Guérin, I. Le Huërou-Luron, R. Thibault, D. Val-Laillet

**Affiliations:** 1 INRAE, INSERM, Univ Rennes, Nutrition Metabolisms and Cancer (NuMeCan), Saint-Gilles, Rennes, France; 2 Department of Digestive Surgery, CHU Rennes, Rennes, France; 3 Nutrition Unit, CHU Rennes, Rennes, France

**Keywords:** Gastrointestinal hormones, Gastrointestinal models, Obesity

## Abstract

Performing the Roux-en-Y gastric bypass (RYGBP) in obese Yucatan minipigs provides an opportunity to explore the mechanisms behind the effects of this surgery in controlled environmental and nutritional conditions. We hypothesized that RYGBP in these minipigs would induce changes at multiple levels, as in obese humans. We sought to characterize RYGBP in a diet-induced obese minipig model, compared with a pair-fed sham group. After inducing obesity with an ad libitum high-fat/high-sugar diet, we performed RYGBP (*n* = 7) or sham surgery (*n* = 6). Oral glucose tolerance tests (OGTT) were performed before and after surgery. Histological analyses were conducted to compare the alimentary limb at sacrifice with tissue sampled during RYGBP surgery. One death occurred in the RYGBP group at postoperative day (POD) 3. Before sacrifice, weight loss was the same across groups. GLP-1 secretion (OGTT) was significantly higher at 15, 30 and 60 min at POD 7, and at 30 and 60 min at POD 30 in the RYGBP group. Incremental insulin area under the curve increased significantly after RYGBP (*p* = 0.02)*.* RYGBP induced extensive remodeling of the alimentary limb. Results show that RYGBP can be safely performed in obese minipigs, and changes mimic those observed in humans.

## Introduction

Obesity prevalence is increasing worldwide. To date, bariatric surgical procedures offer the best opportunity for patients with morbid obesity to obtain long-term weight loss and an improvement in or resolution of their obesity-related comorbidities^1–3^. The Roux-en-Y gastric bypass (RYGBP) is one of the most efficient and widespread bariatric surgery techniques for reducing weight and improving obesity-related diseases, especially type-2 diabetes and cardiovascular disease^4^.

Numerous mechanisms have been put forward to explain weight loss or diabetes resolution, but to date, there is no clearcut hypothesis. The gut-derived peptide hormone glucagon-like peptide 1 (GLP1) acts as an incretin hormone^5^, and is classically assumed to be responsible for the beneficial effects of RYGBP on glucose and energy homeostasis. GLP1 is released by L-cells in response to the presence of nutrients in the gut lumen, and some studies have demonstrated that blocking GLP1 receptors with an agonist (exendin 9–39) reduces β-cell glucose sensitivity and insulin secretion after RYGBP^6,7^. Others have demonstrated that inhibiting the gut hormone response in humans with octreotide restores the food intake previously attenuated by RYGBP^8^. Finally, in rodents, glucose tolerance after RYGBP has been found to vary according to GLP1 agonist (exendin-4) sensitivity^9^. These studies seem to indicate that GLP1 plays an important role in glucose homeostasis after RYGBP. The gut dysbiosis reported in obesity^10^ may improve at the same time as metabolic status and weight loss, suggesting that the gut microbiota is one of the most relevant factors for improvement. Surprisingly, the bariatric procedure only partially restores microbial gene richness, even though major metabolic improvement and weight loss are observed^10^.

To our knowledge, one of the foremost hypotheses is that gut-brain communication is the key to understanding the profound modifications that take place after RYGBP^11–13^. Some human studies have reported changes in brain signaling induced by obesity and their reversal after RYGBP, but the mechanisms are still unclear^11^. One animal study described a diet-induced obesity model in the minipig, where weight gain was 60% higher than the theoretical weight, a signature of morbid obesity. Changes in basal brain metabolism were observed and these changes were similar to those described in obese humans^14^.

Experimental models are needed to decipher the complex mechanisms behind these brain functional changes, which in turn lead to changes in metabolism and behavior. The minipig model represents a prime opportunity for describing and explaining the gut-brain interactions that occur after RYGBP. The pig is one of the closest models to humans, in terms of nutrition physiology, metabolism, and neurobehavior^15–18^. To our knowledge, the first demonstrations of RYGBP in the pig model took place in the 1990s^19,20^. Since then, numerous studies have sought to describe the impact of RYGBP on gastrointestinal morphology, changes in adipose tissue distribution, β-cell and biliary functions, and glucose and incretin regulation^21–26^. However, most studies of RYGBP have so far used growing conventional pigs or normal-weight minipigs, thus reducing the scope and meaning of the results in terms of translational perspectives^23,25,27–29^. Long-term weight loss and reduced food intake have been described in super-obese minipigs, but physiological and metabolic explorations have so far been lacking^30^. Animal models make it possible to design a relevant control group in order to dissociate the respective effects of weight loss and RYGBP procedure. Contrary to studies in humans, animals that have undergone bariatric surgery such as RYGBP can be compared with pair-fed animals that have undergone similar weight loss induced by surgery. Weight loss in itself has beneficial effects on physiological and metabolic markers, which is an important bias for the evaluation of any specific effects and benefits of surgery in a study with no control group. We therefore deemed that before undertaking research on brain signaling and the neurobehavioral impacts of metabolic surgery (i.e. RYGBP), it was essential to assess the feasibility and safety of RYGBP in obese Yucatan minipigs, and to provide metabolic correlates. This would allow us to confirm (or not) that this model can display the metabolic profiles and outcomes observed in humans. Previous descriptions, both of humans and of animal models (rodents or lean pigs), had indicated that glucose homeostasis (including GLP1 response) and the fermentative activity of the intestinal microbiota are important for describing and validating a new animal model. We therefore conducted a pilot study to characterize the RYGBP model, in terms of safety and metabolic effects, in diet-induced obese Yucatan minipigs, comparing them with a pair-fed sham group.

## Results

### Postoperative survival and weight loss

**Figure 1:**
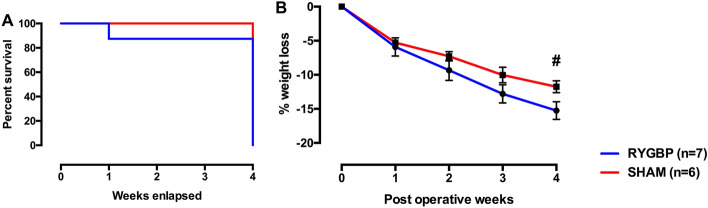
Postoperative animal survival (**A**), and percentage of weight loss after surgery, mean ± *SEM* (**B**). ^#^*p* = 0.08. *RYGBP* Roux-en-Y gastric bypass surgery, *Sham* sham surgery.

A total of 13 obese minipigs underwent surgery (RYGBP: *n* = 7; sham: *n* = 6). One death occurred in the RYGBP group at postoperative day (POD) 3, owing to a massive septic shock induced by a gastrojejunal leak (Fig. 1A). All the other animals survived the postoperative observation period and were euthanized 1 month after surgery. Another animal experienced a late gastrojejunal stenosis with a blind fistula, confirmed both by endoscopy and at euthanasia. Endoscopy was performed because of late recurrent vomiting without strong limitation of the daily food intake. Before surgery, median weight was 82.6 (74.4–88.8) kg. At 1 month, the percentage of weight loss tended to be lower in the RYGBP group than in the sham one (*p* = 0.08) (Fig. 1B). We observed an anorexic phase during the first 5 days after surgery in both groups.

### RYGBP induced a significant increase in GLP1 and insulin secretion

**Figure 2:**
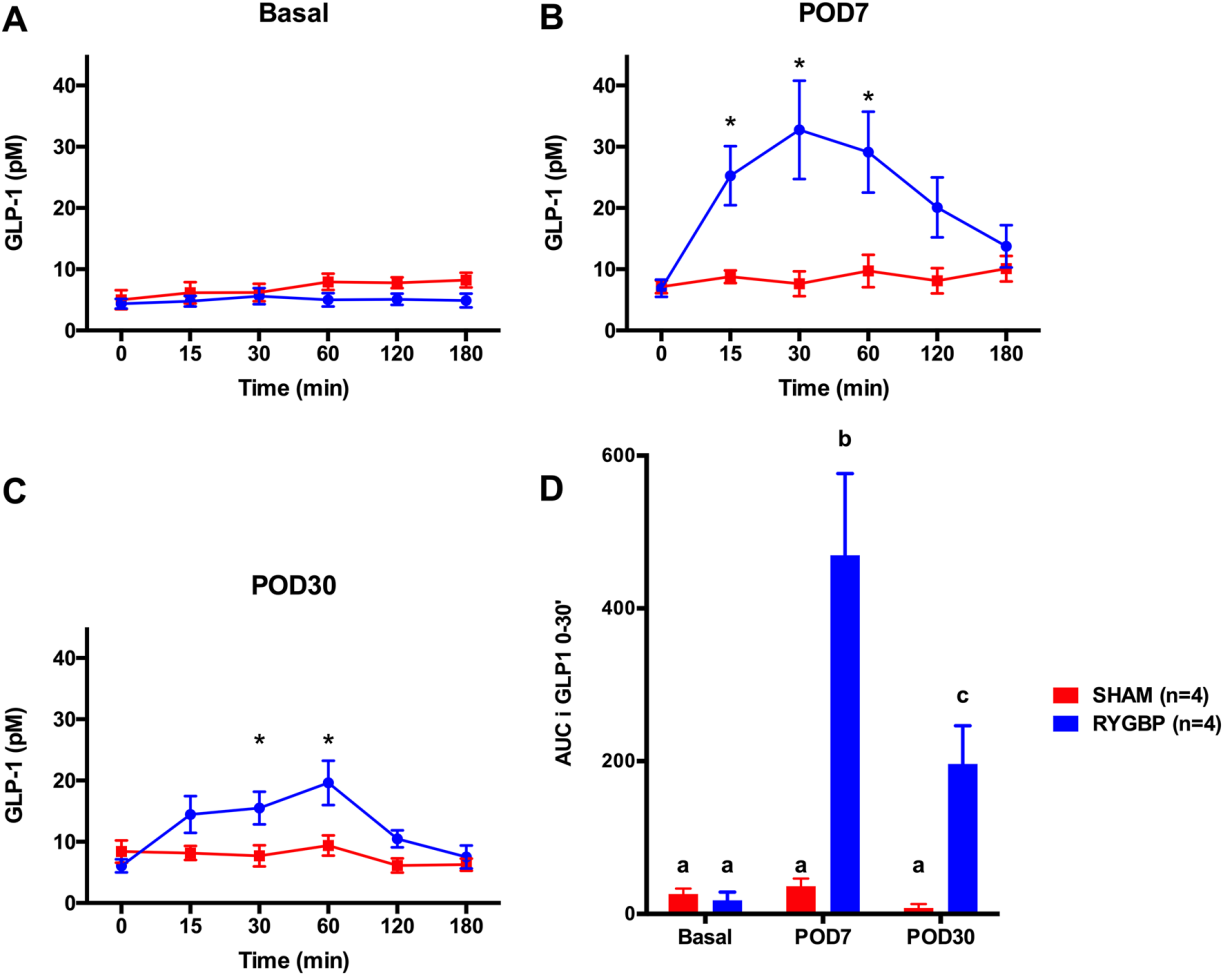
OGTT results before surgery (preoperative, baseline) (**A**), and at postoperative day (POD) 7 (**B**) and POD 30 (**C**). *p < 0.05. Incremental area under the curve (AUCi) of GLP-1 secretion 0–30 min after glucose ingestion (**D**). Letters indicate a significant statistical difference at *p* < 0.05. All values are expressed as means ± *SEM*.

GLP-1 secretion did not differ preoperatively (Fig. 2A), but was significantly higher at 15, 30, and 60 min at POD 7 (Fig. 2B), and 30 and 60 min at POD 30 (Fig. 2C) in the RYGBP group than in the sham group. The incremental area under the curve (AUCi) of GLP-1 between 0 and 30 min was significantly higher in RYGBP than in sham animals at POD 7 and POD 30 (Fig. 2D, Table 1). Concerning the insulin AUCi (Table 1), there was a significant interaction between time and surgery type (RYGBP vs. SHAM; *p* = 0.02). Compared with the baseline condition, the insulin AUCi of the RYGBP group was significantly higher at POD 7 (*p* = 0.003), and tended to be higher at POD 30 (*p* = 0.06). This was not the case of the sham group. The glucose AUCi of the RYGBP group was higher than that of the sham group, at both POD 7 and POD 30 (Table 1), and higher than in the baseline condition at POD 7 (*p* = 0.003) and POD 30 (*p* = 0.01) (Time × Surgery type interaction, *p* = 0.02).

**Table 1:** Metabolic parameters before and after surgery in RYGBP and sham minipig groups.

Variable	Baseline		POD 7		POD 30		*p* value		
	Sham	RYGBP	Sham	RYGBP	Sham	RYGBP	Surgery	Time	Interaction
**Baseline**									
G_0_ (mmol/L)	4.85 ± 0.05	5.19 ± 0.12	4.82 ± 0.11	4.77 ± 0.13	4.74 ± 0.22	4.69 ± 0.18	0.51	0.13	0.35
I_0_ (µIU/mL)	53.06 ± 6.85	66.61 ± 28.41	59.19 ± 4.20	49.88 ± 9.21	47.19 ± 7.56	49.58 ± 12.72	0.85	0.72	0.72
HOMA-IR	11.43 ± 1.46	15.56 ± 6.81	12.68 ± 0.99	10.62 ± 2.01	10.12 ± 2.05	10.60 ± 3.11	0.76	0.65	0.65
**Oral glucose challenge test (OGTT) **									
Glucose									
AUCG_i0-30_ (mmol/L/30 min)	24.11 ± 5.12	23.80 ± 7.23	25.62 ± 8.82	65.96 ± 4.26	19.08 ± 2.50	57.80 ± 11.39	< 0.01	0.03	0.02
Glucose peak (mmol/L)	5.69 ± 0.20	5.91 ± 0.40	6.13 ± 0.57	7.22 ± 0.48	5.24 ± 0.34	6.54 ± 0.36	0.04	0.09	0.39
**Insulin**									
AUCi_i0-30_ (µIU/mL/30 min)	2307.74 ± 385.64	1279.12 ± 589.87	2064.35 ± 457.75	4170.41 ± 1482.02	2113.94 ± 820.94	3022.55 ± 1324.58	0.6	0.05	0.02
Matsuda index	23.64 ± 1.78	28.91 ± 5.92	20.56 ± 2.39	19.26 ± 3.35	31.45 ± 6.78	26.13 ± 6.01	0.93	0.11	0.43
**GLP-1**									
AUC_i0-30_ (pmol/L/30 min)	26.12 ± 7.19	17.73 ± 11.21	36.30 ± 10.30	469.60 ± 107.23	7.92 ± 4.67	196.43 ± 50.44	< 0.01	< 0.01	< 0.01
**Lipid profile**									
Free fatty acids (mmol/L)	0.67 ± 0.12	0.44 ± 0.18	0.84 ± 0.21	0.66 ± 0.08	0.61 ± 0.09	0.40 ± 0.12	0.22	0.10	0.96
Triglycerides (mmol/L)	0.45 ± 0.05	0.38 ± 0.06	0.64 ± 0.09	0.60 ± 0.13	0.54 ± 0.14	0.36 ± 0.02	0.3	0.06	0.67
Total cholesterol (mmol/L)	2.29 ± 0.10	2.52 ± 0.21	2.01 ± 0.09	1.92 ± 0.15	1.95 ± 0.14	1.86 ± 0.06	0.91	< 0.01	0.28
HDL(mmol/L)	0.83 ± 0.08	0.95 ± 0.10	0.52 ± 0.03	0.51 ± 0.03	0.62 ± 0.06	0.52 ± 0.03	0.95	< 0.01	0.24
LDL(mmol/L)	1.26 ± 0.15	1.40 ± 0.21	1.19 ± 0.13	1.13 ± 0.13	1.08 ± 0.14	1.18 ± 0.09	0.73	0.19	0.63
**Inflammation**									
Haptoglobin (g/L)	3.77 ± 0.46	4.42 ± 0.60	4.44 ± 0.35	4.43 ± 0.24	2.42 ± 0.34	2.92 ± 0.36	0.37	< 0.01	0.64

### Impact of weight loss on systemic inflammation and lipid profile

Haptoglobin was significantly lower at POD 30 than at baseline or POD 7 in both groups (RYGB: *p* = 0.03 and *p* = 0.03; sham: *p* = 0.05 and *p* = 0.005), which did not differ from each other (*p* = 0.37) (Table 1). Total cholesterol was significantly lower at POD 7 and POD 30 than at baseline in the RYGBP group (*p* = 0.005 and *p* = 0.002), but there was no effect of type of surgery. High-density lipoprotein (HDL) cholesterol was significantly lower at POD 7 and POD 30 than at baseline in the RYGBP group (*p* = 0.008 and *p* = 0.001), and lower at POD 7 in the sham group (*p* = 0.01 and *p* = 0.08), but there was no significant effect of type of surgery.

### Impact of weight loss and food restriction on fecal SCFA content

**Figure 3:**
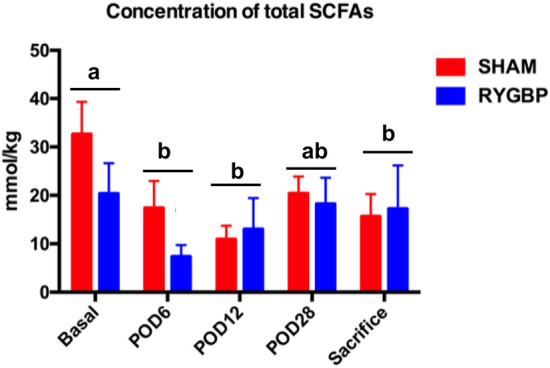
Total concentration of short-chain fatty acids (SCFAs) before surgery (baseline), at postoperative days (PODs) 6, 12 and 28, and at sacrifice (POD 30). *RYGBP* Roux-en-Y gastric bypass surgery, *Sham* sham surgery. Time effect: *p* = 0.002*.* There was no effect of type of surgery (sham vs. RYGB) at any time. Different letters indicate a significant statistical difference at *p* < 0.05.

Quantitative analysis of total short-chain fatty acids (SCFAs) after surgery showed no significant difference between groups (surgery type effect, *p* = 0.47) (Fig. 3), but both RYGBP and sham surgery induced a decrease in intestinal SCFA concentrations (time effect, *p* = 0.02) between POD 6 and sacrifice (*p* < 0.05 at POD 6, POD 12 and sacrifice, but *p* = 0.08 at POD 28).

### RYGBP-induced remodeling of alimentary limb

**Figure 4:**
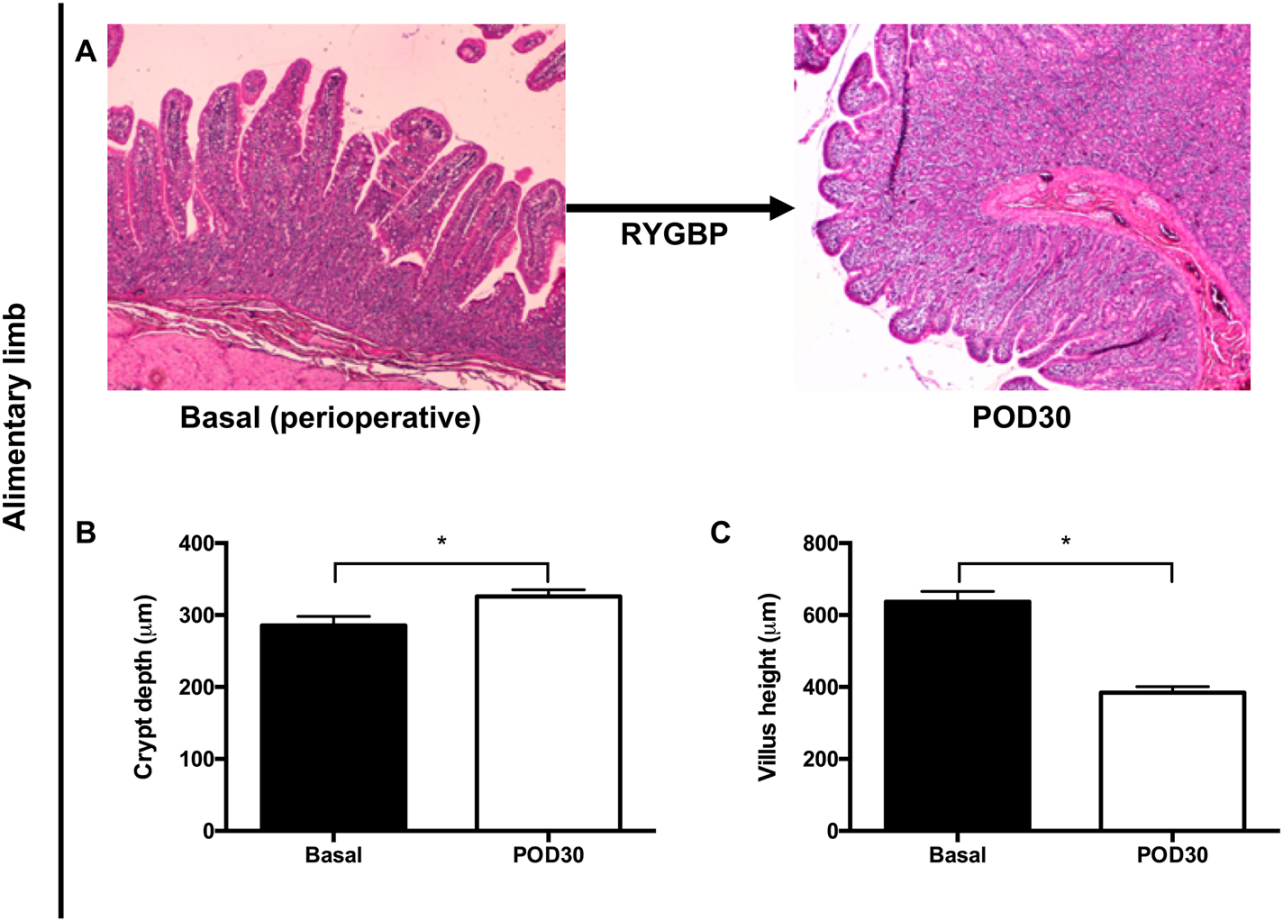
Remodeling of the alimentary limb before (preoperative) and after surgery at postoperative day (POD) 30 in the RYGBP group (**A**). Changes in crypt depth (**B**) and villus height (**C**). All values are expressed as mean ± *SEM*. *p < 0.05. *RYGBP* Roux-en-Y gastric bypass surgery.

RYGBP induced a significant remodeling of the alimentary limb (Fig. 4A). Compared with the preoperative baseline condition, there was an increase in jejunal crypt depth at POD 30 (325 ± 9.46 vs. 285.42 ± 12.65 µm (baseline), *p* = 0.01) and a decrease in villus height at POD 30 (384.45 ± 16.42 vs. 637.00 ± 28.54 µm (baseline), *p* < 0.01) (Fig. 4B,C).

## Discussion

The present pilot study showed that the development of an obese minipig model of RYGBP, for which data were lacking in the literature, is feasible and that the metabolic response in terms of glucose sensitivity and GI tract histological changes are close to those observed in humans. Thanks to the reproducibility of our model, and the close similarities between minipig and human in terms of anatomy, physiology and brain functions, we are convinced that this is a highly relevant model for further research aimed at deciphering the microbiota-gut-brain mechanisms underlying the beneficial metabolic outcomes of RYGBP in humans.

Models of bariatric surgery in obese minipigs are scarce, and to our knowledge, ours is the first report of an obese Yucatan minipig model of RYGBP documented with metabolic measurements. Among the seven RYGBP procedures we conducted, we only observed one death (massive septic shock arising from gastrojejunal leak). This animal was the first to be operated, and the gastrojejunal anastomosis technique was improved for the subsequent pigs. As a result, no other lethal or severe postoperative complications were observed, although there was one case of anastomotic stenosis with a blind fistula, confirmed by upper-GI endoscopy. Some teams have reported high postoperative complications or a high death rate, both attributable to anesthesia management and fistulas^19,31^. Thanks to the recognized experience of our laboratory in terms of surgical procedures in minipigs, especially obese ones, RYGBP was conducted safely. It should be noted that we encountered difficulties with gastric pouch stapling. In contrast to the lean minipig model, we were confronted with very thick stomach walls that complicated the task of safely stapling the gastric pouch. These difficulties explained-at least in part-the death reported above, owing to a fragile gastric pouch and, consequently, a fragile gastrojejunal anastomosis. Stapling was still difficult even when the thickest commercially available staplers were used (Johnson & Johnson Echelon Flex stapler with black reload) and this situation was encountered in all operated cases. Despite these technical difficulties, we continued to construct the classic gastric pouch (i.e. 30 mL), in order to reproduce the RYGBP procedure as faithfully as possible, thereby minimizing biases and facilitating the translation of our results for human health.

We chose to adopt an open surgical approach, rather than a laparoscopic approach. First, even with an open approach, reported postoperative outcomes were complicated in a large number of cases^27,32^, and dramatically so in laparoscopic ones^31^. As we aimed to establish a model in an obese condition to conduct longitudinal assessments in further protocols (i.e. over several months), we preferred to choose the safest intervention, to limit the risk of postoperative complications.

Interestingly, weight loss percentage at 1 month tended to be higher in the RYGBP group, even though daily food intake was identical in terms of energy composition in both groups. Owing to the limited number of animals included in this pilot study, the significance threshold was not reached, and there was probably insufficient statistical power. Results published by Cavin et al. in a rodent model^33^ indicated a clear difference in body weight loss percentages between the sham and RYGBP groups (no statistics reported). However, higher food intake during the first 20 days, with “free access to solid normal diet” (p. xx) in both groups induced an inevitable bias in assessing the specific effect of RYGBP.

To validate our model, we explored hormonal changes induced by RYGBP surgery, as previously described in animal models and humans. GLP-1 secretion had previously been reported by other teams to dramatically increase in the lean pig model^26,27^ and in humans^8^ after RYGBP. Interestingly, we also found a significant increase in plasma GLP-1 concentration at POD 7 and POD 30. The substantial GLP-1 response to oral glucose tolerance tests (OGTTs) after RYGBP is thought to be involved in type-2 diabetes remission after RYGBP^25^, but this hypothesis was refuted by Mokadem et al.^34^, who reported similar responses to RYGBP in both wild-type mice and two nonfunctional GLP-1 mice (α-gustidin deficient and GLP1-receptor deficient). As with the improvement in fasting glucose independent of weight loss, which has also been observed after sleeve gastrectomy^35^, an increase in GLP-1 is probably neither necessary nor the determining factor for the improvement in glucose homeostasis observed after bariatric surgery, and certainly not over the long term after surgery^7^. The higher insulin AUCi observed in the RYGBP group in response to an OGTT was in line with previous studies^22,25^, and consistent with our results on GLP-1 secretion. The major increase in the blood glucose AUCi observed after RYGBP can be explained by accelerated gastric emptying in the RYGBP group (30-mL gastric pouch and 257 mL mean volume of ingested glucose solution), and may have biased insulin secretion and GLP1 assessment. However, the non-different global AUC of blood glucose (data non shown) also in line with previous published data^26^ allows us to conclude on the global secretion of GLP-1 and insulin. Previous studies had reported a reduced GLP-1 response 1 year after surgery^36^. Here, we observed reduced GLP-1 secretion after OGTT at POD 30, compared with POD 7. Several possible explanations for this reduction have been put forward, such as a faster process in pigs than in humans, with a decreased response already observable at 1 month, or a weight-related cause^26^, but to date, none has proved sufficiently robust.

When Lindqvist et al.^26^ assayed the active GLP-1 response to OGTT, they unexpectedly found that in lean pigs, active GLP-1 was lower in the RYGBP group than in the sham one. The authors suggested that increased DPP-4 inhibitor activity and a more rapid degradation of GLP-1 after RYGBP could explain those findings. Accordingly, even though our results allowed us to validate our model, the important question of the true role of GLP-1 in the remission of type-2 diabetes after surgery has yet to be answered.

Another interesting result of our study was the profound remodeling of the alimentary limb, with increased crypt depth and decreased villus height, as observed after RYGBP in humans^33^. It is interesting to note that Cavin et al. obtained different results with their rodent model concerning villus height. They observed a significant increase in villus height, in contrast to our study, and more importantly, in contrast to the remodeling observed in humans, which reinforces the relevance of our minipig model to human physiology.

Decreased fermentative activity was observed in both groups from POD 6 onwards, in line with previously published dat^37,38^a. The equal quality and quantity of food intake in the two groups may explain the absence of differences in fecal SCFA levels, as decreased SCFA concentrations are mainly observed in patients who have undergone bariatric surgery when their subsequent carbohydrate intake is reduced^37^. Even if there was no difference between the two groups, the quantitative measurements of SCFAs used to assess microbiota activity did not explore differences in the composition and abundance of the microbiota. The microbiota seems to be an important factor in metabolic resolution after obesity surgery^13^, but the results in the literature remain inconsistent, probably because the control group is not always the most appropriate one (i.e. frequently baseline state or lean human as control) for exploring the specific effect of RYGBP^13,37,38^ on intestinal microbiota.

Our study obviously had several limitations. The number of animals we operated on was low, but still reasonable for a feasibility and pilot study with a real control group (i.e. sham surgery with food restriction equal to RYGBP group, following a pair-feeding design). Because of the limited number of animals and the high intrinsic variability of some measurement methods, we encountered high interindividual variability in the baseline levels of glucose and insulin, for example. However, we applied a strong statistical analysis plan with systematic two-way analyses of variance (ANOVAs) and Tukey post hoc tests for repeated measures, thereby limiting possible misinterpretations.

## Conclusion

This pilot study demonstrated for the first time that RYGBP can be safely and successfully performed in obese minipigs. Comparison with a pair-fed sham group allowed us to identify metabolic and physiological outcomes that were specific to the RYGBP group (i.e. independent of weight loss), and consistent with observations in humans concerning the remodeling of the alimentary limb and GLP1/insulin secretion in response to oral glucose tolerance test. Further studies are needed to use this RYGBP model to carry out detailed investigations of the gut-brain mechanisms leading to the modulation of brain function and behavior.

## Methods

### Animals and experimental design

The experiments described here were conducted at the INRAE center of Saint-Gilles (agreement no. 3527532) in France, in accordance with the current ethical standards of the European Community (Directive 2010/63/EU). The Brittany Regional Ethics Committee on Animal Experimentation validated and approved the entire procedure described here, and the final authorization was obtained from the French Ministry of Higher Education, Research and Innovation (Project APAFIS#598-201504280924565 v5). The study was also carried out within ARRIVE guidelines.

Animals were housed in individual pens measuring 110 × 80 cm and 110 cm high, with bars allowing contact between animals in adjacent pens. They were kept in a 50 m^2^ room in controlled conditions: the temperature was maintained at around 22 °C and the room was equipped with artificial lighting for a 15:9 h light–dark cycle. Cages were equipped with individual 50-cm-long troughs, each animal had free access to water, and pens were equipped with a metal chain to play with.

Before induction of obesity, and as routinely done in our department, animals were fed a standard diet of 0.30 MJ/kg body weight^0.75^/day. Obesity was induced in 11 lean adult Yucatan minipigs with an obesogenic diet consisting of an ad libitum high-fat and high-sugar diet (10.8 MJ/kg) (Supplementary Table 1) for at least 6 weeks^14^. To perform further blood sampling, a central venous catheter was implanted in all animals under general anesthesia 2 weeks before RYGB surgery. Obese minipigs were then randomly assigned to the RYGBP (*n* = 7) or sham surgery (*n* = 6) groups. OGTTs were performed before (baseline OGTT), and 1 week (POD 7 OGTT) and 1 month (POD 30 OGTT) after surgery. Feces samples were collected manually in the rectum before surgery, at POD 6, 12 and 28, and at sacrifice. A jejunum segment (4–5 cm section removed 70 cm from the Treitz ligament) was systematically sampled for histological analysis during surgery (RYGBP group only).

One month after surgery, all minipigs were euthanized by intravenous injection of T-61 (MSD Santé Animale, Beaucouze, France) under anesthesia. After euthanasia, a prompt laparotomy was performed, and a short jejunum segment from the distal part of the alimentary limb just above the jejuno-jejunal anastomosis was resected (RYGBP group only).

### Bariatric surgery: anesthesia, surgery and postoperative care

All surgeries were conducted under general anesthesia by laparotomy after an overnight fast. Even though our INRAE surgical platform is equipped with a laparoscopic column, and our surgeon (DB) for this study is a well-trained senior surgeon who classically uses laparoscopy for RYGBP in humans, laparotomy was preferred over laparoscopy. This choice was made in order to reduce interindividual variability during the surgical procedure and maximize the success of surgery. The minipig intestine is sometimes more fragile than the human one, whereas the stomach has a thicker wall, making the stapling procedure more difficult. Anesthesia was induced by an intramuscular injection of ketamine 5 mL/kg (Imalgene 1000; Merial, Lyon, France) and maintained during surgery with isoflurane inhalation (AErrane 100 mL; Baxter SAS, France). Artificial ventilation was performed after tracheal intubation with a frequency of 15 breaths/min, tidal volume of 420–470 mL, and spCO_2_ below 5%. Pain management was ensured with intravenous injection of fentanyl (Renaudin, Itxassou, France) 50 μg/mL at 0.4 mL/min. Vascular filling was ensured with a Ringer Lactate (Braun Medical) drip at 22 mL/min.

All surgeries were performed by DB. A classic RYGBP was conducted to reproduce the human model with a 150-cm alimentary limb, a 70-cm biliary limb, and a small gastric pouch (30 mL) with a calibrated gastrojejunal anastomosis, as described in Verhaeghe et al.^27^. Both gastrojejunal and jejunojejunal anastomoses were hand-sewn with a 4.0 absorbable running suture. Mesenteric defects were systematically closed. In the sham group, a laparotomy was performed and the intestine was handled for at least 20 min, before closing the laparotomy.

After surgery, pain management was ensured by subcutaneous injection of morphine hydrochloride (Renaudin) 0.5 mg/kg/BW. Antibioprophylaxis using intramuscular injection of amoxicillin (Duphamox LA; Zeotis, Paris, France) 15 mg/kg BW was performed the day of the surgery and 2 days later.

Diet recovery was adapted from the postoperative protocol used in humans who have undergone RYGBP. Water intake was allowed the night of the surgery, and an exclusive mixed standard diet was offered for 3 weeks, before returning to a standard diet (7.27 MJ/kg) based on feed pellets. The reintroduction of food after surgery was done gradually to reach the objective (500 g/day; 3.635 MJ/day) at POD 5. The 500 g/day ration was maintained in both groups during the postoperative period, to avoid the amount ingested influencing the assessment of the metabolic consequences of surgery. It took approximately 1 week to reach the daily feed ratio objectives (500 g/day) in both groups. The pigs were monitored on a daily basis by staff members, to assess their general condition, pain control, and food tolerance. All animals were weighed once a week.

### Oral glucose challenge tests

OGTTs were performed after an overnight fast. Each one consisted of the administration of 1 g/kg glucose (constituted according to the animal’s weight at the time of the OGTT with a glucose 30% (B. Braun) solution; i.e. mean ± standard error of the mean (*SEM*) = 257 ± 8.8 mL) through a tube inserted in the mouth of the animal. Glucose solution was naturally swallowed. Blood sampling (5 mL) was performed before glucose ingestion (t0), and after 5, 15, 30, 60, 120 and 180 min. Blood was collected in tubes containing K2-EDTA for glucose, insulin, inflammatory marker and lipid profile, and tubes containing K2-EDTA plus an anti-DPP-4 (Millipore, Billerica, MA, USA), 10 µL/mL of blood, for GLP-1. After centrifugation at 2500*g* for 10 min at 4 °C, the plasma was immediately collected and stored at − 80 °C for GLP1 analysis, and at − 20 °C for glucose, insulin, inflammatory marker and lipid profile analyses.

### Hormone, glucose, lipid and inflammatory marker plasma assays

As described in a previous study^39^, plasma glucose, non-esterified fatty acids, triglycerides, total cholesterol, HDL cholesterol and haptoglobin were measured using an automated spectrophotometric method (Konelab 20i; Thermo Fisher Scientific, Illkirsh, France) and specific commercial kits (bioMérieux, Bruz, France). The intra-assay coefficient of variation (CV) was < 5%.

GLP-1 concentrations were measured using an active GLP-1 ELISA kit (Millipore). Insulin concentrations were measured using a radioimmunoassay (RIA) method, with iodinated porcine insulin as a tracer (INSULIN-CT; CIS Bio International, Gif sur Yvette, France). The intra- and inter-assay CVs were 15% and 11%, for a concentration of 35 µIU/mL.

### Fermentative activity assessment

Fermentative activity of the intestinal microbiota was assessed through the quantitative analysis of SCFA in feces. Samples of feces were stabilized with 0.5% orthophosphoric acid at a rate of 1 mL acid/g feces. After centrifugation at 1700*g* for 15 min at 4 °C, 1 mL of supernatant by sample was stored at − 20 °C before SCFA measurement using gas chromatography analysis, as described elsewhere^39^.

### Histological analyses

In line with a previous study^40^, jejunal segments were emptied and fixed in 4% paraformaldehyde for 24 h at 4 °C until serial dehydration in ethanol and embedding in paraffin for further morphometry analysis. Villus length and crypt depth were determined on hamatoxylin-eosin-stained jejunal sections (5 µm). A minimum of 15–20 well-oriented crypt-villus units were measured under a light microscope (Nikon Eclipse E400; Nikon Instruments, France) using image analysis software (ImageJ, version IJ1.44). All morphological measurements were conducted by the same investigator (MF), who was blinded to the minipig group from which each section was taken.

### Statistical analysis

All analyses were performed using the open-source R statistical software (R: A Language and Environment for Statistical Computing; R Core Team, R Foundation for Statistical Computing, Vienna, Austria; https://www.r-project.org/). Quantitative variables were expressed as medians with interquartile range or as means with *SEM*. For simple comparisons, we ran nonparametric Mann–Whitney U tests. We ran Type-III ANOVAs to test the Surgery type × Time interaction with post hoc Tukey tests for repeated measures. Figures were generated with GraphPad Prism. The significance threshold was set at *p* < 0.05, and a trend was considered when 0.05 < *p* < 0.1.

### Ethical approval

The Brittany Regional Ethics Committee on Animal Experimentation validated and approved the entire procedure described in this work, and the final authorization was obtained from the French Ministry of Higher Education, Research and Innovation (Project APAFIS#598-201504280924565 v5). The experiments described in this paper were conducted at the INRAE center of Saint-Gilles (agreement no. 3527532) in France, in accordance with the current ethical standards of the European Community (Directive 2010/63/EU). No experimentation on humans and/or the use of human tissue samples were conducted in the current study.

## Supplementary Information

Supplementary Information.

## Data Availability

Datasets generated, analyzed and described in this paper will be made available upon reasonable request. Requests for access should be addressed to the corresponding author: damien.bergeat@chu-rennes.fr.

## Abbreviations

RYGBP: Roux-en-Y gastric bypass
GLP1: Glucagon-like peptide 1
OGTT: Oral glucose tolerance test
POD: Postoperative day
HDL: High-density lipoprotein
SCFA: Short-chain fatty acid
SEM: Standard error of the mean
AUCi: Incremental area under the curve
