# Supplementary material for: A pilot study about the development and characterization of a Roux en Y gastric bypass model in obese Yucatan minipigs
Source: Sci Rep. 2021 Oct 12;11:20190. doi: 10.1038/s41598-021-98575-8 (PMC8511153; doi:10.1038/s41598-021-98575-8)
Supplement: Supplementary file 1 — Supplementary Information. [file 41598_2021_98575_MOESM1_ESM.docx]

**A pilot study about the development and characterization of a Roux en Y gastric bypass model in obese Yucatan minipigs**

Bergeat, D.^1,2*^, Blat, S.^1^, Gautier, Y.^1^, Guérin, S.^1^, Le Huërou-Luron, I.^1^, Thibault, R.^1,3$^, Val-Laillet, D.^1$^

**Supplementary Table 1** Obesogenic high-fat and high-sugar diet composition.

| **Composition (%)** | | **High-fat sucrose (HFS)** | **Standard (STD)** |
| --- | --- | --- | --- |
| **Wheat** | | 6.25 | 10 |
| **Barley** | | 12 | 33 |
| **Wheat bran** | | 14 | 25 |
| **Soybean meal** | | 12 | 6 |
| **Sunflower meal** | | 8 | 10 |
| **Soybean hulls** | | 8 | 12 |
| **Molasses** | | - | 1 |
| **Corn starch** | | 6.5 | - |
| **Sucrose** | | 20 | - |
| **Lard oil** | | 10 | - |
| **Bicalcium phosphate** | | 0.6 | 0.6 |
| **Calcium carbonate** | | 1.3 | 1.3 |
| **NaCl** | | 0.6 | 0.6 |
| **Mineral Vitaminic Complement** | | 0.75 | 0.5 |
| **Total** | | 100 | 100 |
| **Metabolizable energy (MJ/kg)** | | 14.09 | 10.31 |
| **Net energy (MJ/kg)** | | 10.8 | 7.27 |
|  |  |  |  |
| **Nutritional value (%)** | |  |  |
| **Dry matter** | | 91.8 | 87.7 |
| **Cellulose** | | 7.49 | 11.05 |
| **Glucids (starch)** | | 38.34 | 28.23 |
| **Lipids** | | 11.38 | 2.17 |
| **Nitrogen matter** | | 12.74 | 15.22 |
| **Mineral content** | | 5.97 | 6.81 |
